# OTG-snpcaller: An Optimized Pipeline Based on TMAP and GATK for SNP Calling from Ion Torrent Data

**DOI:** 10.1371/journal.pone.0097507

**Published:** 2014-05-13

**Authors:** Pengyuan Zhu, Lingyu He, Yaqiao Li, Wenpan Huang, Feng Xi, Lin Lin, Qihuan Zhi, Wenwei Zhang, Y. Tom Tang, Chunyu Geng, Zhiyuan Lu, Xun Xu

**Affiliations:** 1 BGI-Shenzhen, Shenzhen, China; 2 Complete Genomics, Inc., Mountain View, California, United States of America; Dana-Farber Cancer Institute, United States of America

## Abstract

Because the new Proton platform from Life Technologies produced markedly different data from those of the Illumina platform, the conventional Illumina data analysis pipeline could not be used directly. We developed an optimized SNP calling method using TMAP and GATK (OTG-snpcaller). This method combined our own optimized processes, Remove Duplicates According to AS Tag (RDAST) and Alignment Optimize Structure (AOS), together with TMAP and GATK, to call SNPs from Proton data. We sequenced four sets of exomes captured by Agilent SureSelect and NimbleGen SeqCap EZ Kit, using Life Technology’s Ion Proton sequencer. Then we applied OTG-snpcaller and compared our results with the results from Torrent Variants Caller. The results indicated that OTG-snpcaller can reduce both false positive and false negative rates. Moreover, we compared our results with Illumina results generated by GATK best practices, and we found that the results of these two platforms were comparable. The good performance in variant calling using GATK best practices can be primarily attributed to the high quality of the Illumina sequences.

## Introduction

With the development of DNA sequencing technology, high-throughput sequencing has been widely used in the life sciences. However, the considerable price of a sequencing machine and the long time needed to perform sequencing are substantial obstacles preventing this sequencing technology from being applied in the clinical field. To overcome these two shortcomings, Roche 454, Illumina and Life Technologies released benchtop personal genome machines, which are the 454 GS Junior, the MiSeq and the Ion Torrent PGM/Proton, respectively [1], [2]. In comparison with previous editions, both the sequencing times and the handling procedures have been greatly reduced, making these machines suitable for use in a hospital setting. Ion Torrent PGM, the first benchtop sequencing machine, is an Ion-based sequencing machine that was designed by Rothberg and released in February 2010. Due to the lack of a laser light, CCD imaging or fluorescence labeling, the cost of Ion Torrent sequencing is relatively low. Because the Ion Torrent PGM was mainly designed for small target region or cancer panel sequencing, Life Technologies updated PGM to Proton, which specializes in RNA and exome sequencing.

Although sequencing technology is popular and therefore quickly updated, sequencing the entire genome remains costly. One technique to remedy this problem is to isolate the target region of interest before sequencing. Applying this strategy greatly reduces the cost of sequencing, allowing researchers to focus on the region of interest instead of the whole genome. Currently, Agilent SureSelect, NimbleGen SeqCap and Illumina TruSeq are the main sequence capture methods for next generation sequencing [3], [4].

There are many differences between Ion Torrent and Illumina platform. Ion Torrent only produces single-end (SE) reads that vary in length. However, Illumina can produce both SE and paired-end (PE) reads with fixed read lengths. Therefore, these two platforms employ different duplication removal algorithms. Another difference is that Ion Torrent is prone to homopolymer sequencing errors, including overcall and undercall of the number of homopolymer bases [5], [6], [7], [8], [9]. We aligned the Ion Torrent reads to the reference and counted the number of gaps using samtools [10]. There was1 gap every 100 bp on average, which is much more frequent than the Illumina data. Therefore, the method to detect variants in Ion Torrent reads must be improved.

## Results

**Figure 1:**
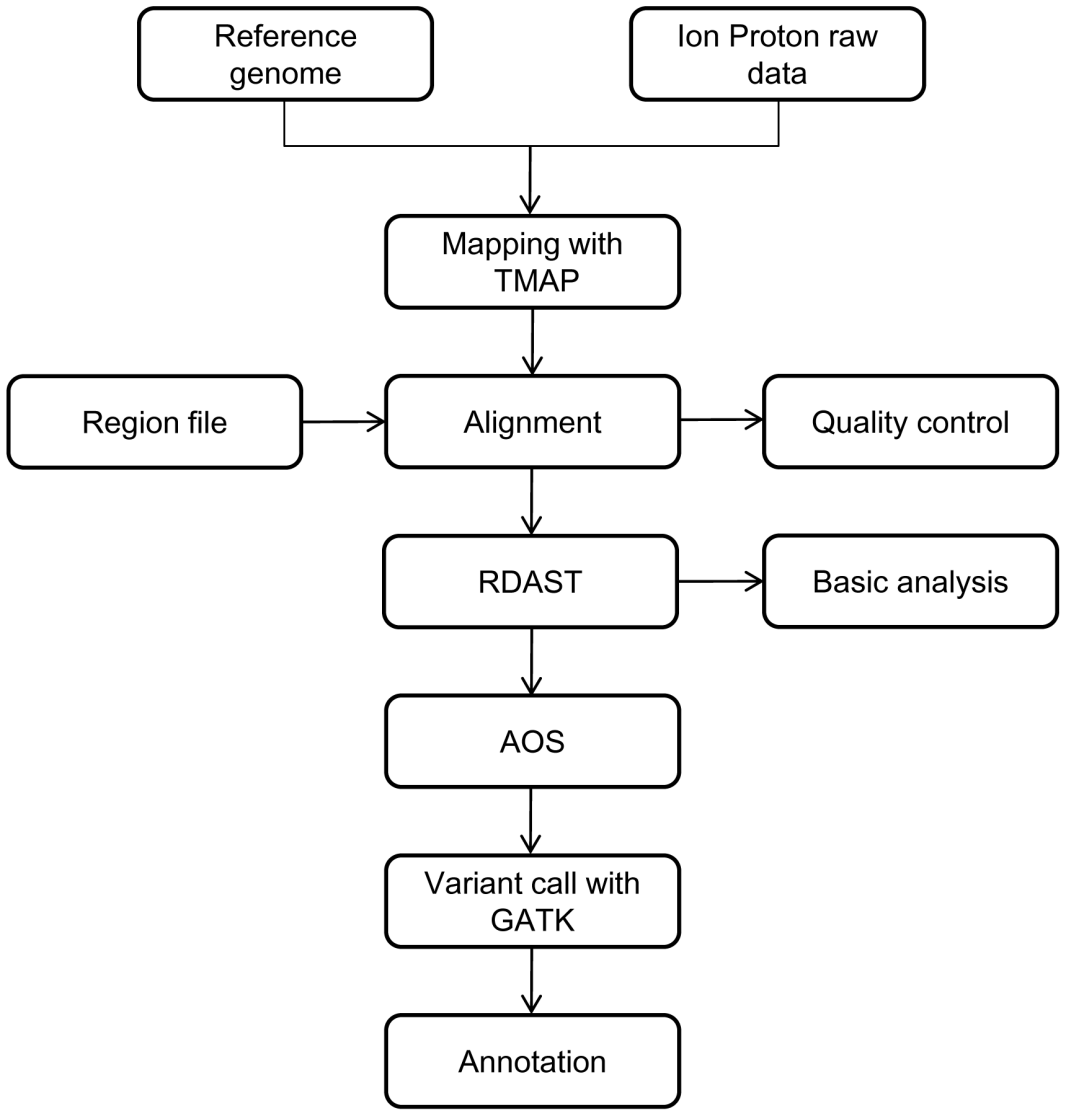
Pipeline of OTG-snpcaller.

We developed a pipeline, known as the optimized pipeline, based on TMAP and GATK (**OTG-snpcaller**) to detect SNPs using data from the Ion Torrent Proton platform. Figure 1 describes the flow chart of this pipeline. The raw data, in bam format, were generated from the Proton sequencer after the trim and filter processes, which were carried out using Torrent Suite Software (TSS) v3.6 (http://ioncommunity.lifetechnologies.com/docs/DOC-7463) Then, TMAP3.6 (https://github.com/iontorrent/TMAP) was selected to perform the alignment and generate the results, which were also in bam format. After removing the duplicates and performing structural optimization of the alignment result, GATK was performed on the detected variants [11]. Then, we compared the results of OTG-snpcaller with those of Torrent Variant Caller (TVC) v3.6 (http://ioncommunity.lifetechnologies.com/docs/DOC-8367) and the Illumina variant call pipeline to evaluate the performance of our method.

### 1. Establishment of OTG-snpcaller

#### a. Choose alignment software

We aligned the reads of the TP00005 library to HG19 by BWA (version 0.7.5) [12] and TMAP3.6using the default parameters. For BWA-SW, the reads mapping rate was 84.13%, and the effective base rate was 95.97% (Table 1). However, for TMAP, the reads mapping rate was 97.82%, which is much higher than that of the BWA-SW alignment, and the effective rate was 96.55%. Moreover, the run time of TMAP was only 2.5 h, which is much less than that of BWA-SW (39 h). Furthermore, the TMAP mapping quality was more suitable for variant detection. Consequently, we choose TMAP3.6 as our alignment software in OTG-snpcaller.

**Table 1:** Mapping results of the TP00005 library using BWA and TMAP.

Software	Reads map rate	Total map base	Effective base*	Effective base rate	Run time
**BWA-SW**	84.13%	9906.19M	9507.5M	95.97%	39 h
**TMAP3.6**	97.82%	10392.84M	10034.29M	96.55%	2.5 h

*Effective base means the actually mapped bases, not including the soft clipped bases.

#### b. Remove Duplicates according to the Alignment Score Tag (RDAST)

**Figure 2:**
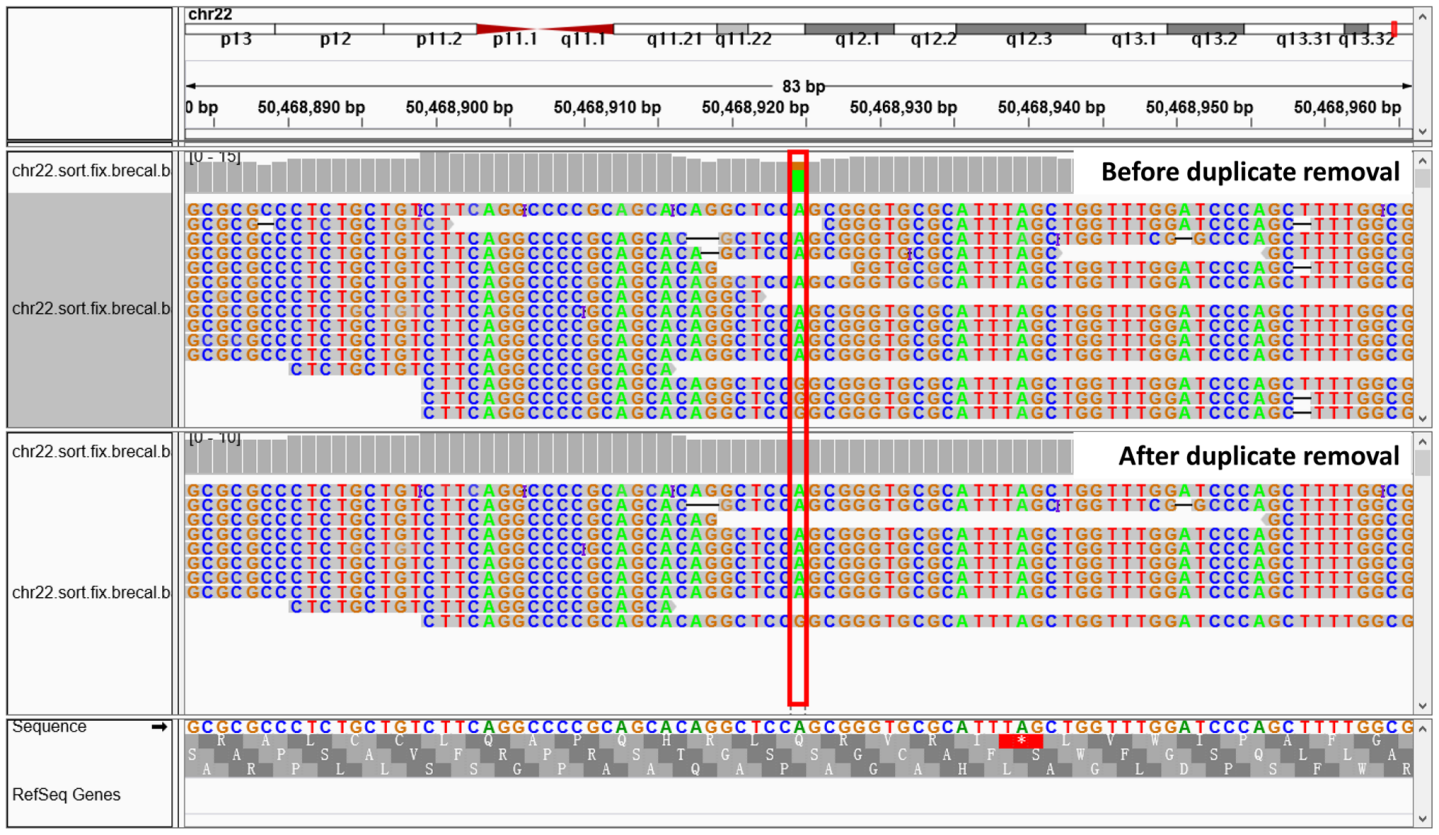
Reads distribution before and after RDAST treatment. The position in the red box, ischr22∶50468925, for which three reads of genotype G make up 27.27% of the results, will show up as a false positive site. However, after RDAST treatment, two reads are deleted, and the remaining read accounts for 12.5% of all reads in this position. Consequently, this false positive result disappears.

Duplicate reads will cause errors in variant detection. For example, the errors introduced by PCR, such as mismatches and gaps, will accumulate in duplicate reads. A false positive variant result is more likely when too many duplicate reads are mapped to the same location in the reference. The allele frequency will be impacted as well, especially when there is low depth (Figure 2). Therefore, we introduced the RDAST (short for Remove Removing Duplicates according to Alignment Score Tag) procedure to solve this problem.

We developed the RDAST computing method specifically for Ion Proton data to remove the duplicate reads. The length of reads may change significantly after carrying out the trimming and filtering steps of the torrent sever, but the location of the 5′ strand in the reference genome is fixed. MarkDuplicates in Picard (http://picard.sourceforge.net/) will only keep one read from the piled-up reads mapped to the same start site. Approximately 50% of the total reads will be deleted using this tool, which seems to be a substantial loss of read data. We decided to use a more flexible approach, which chooses a certain alignment score (AS) as the threshold to filter trash reads. For a fixed reference genome position, RDAST keeps the reads having alignment score (AS) higher than the threshold. Certain parts of the genome that are hard to sequence, such as the repeat regions, contain short reads with very low AS that will gather together. If all of these reads are removed, it would be impossible to detect variations in these regions. Therefore, when there are no reads with an AS higher than the threshold, RDAST keeps one read, the read with the highest AS.

**Figure 3:**
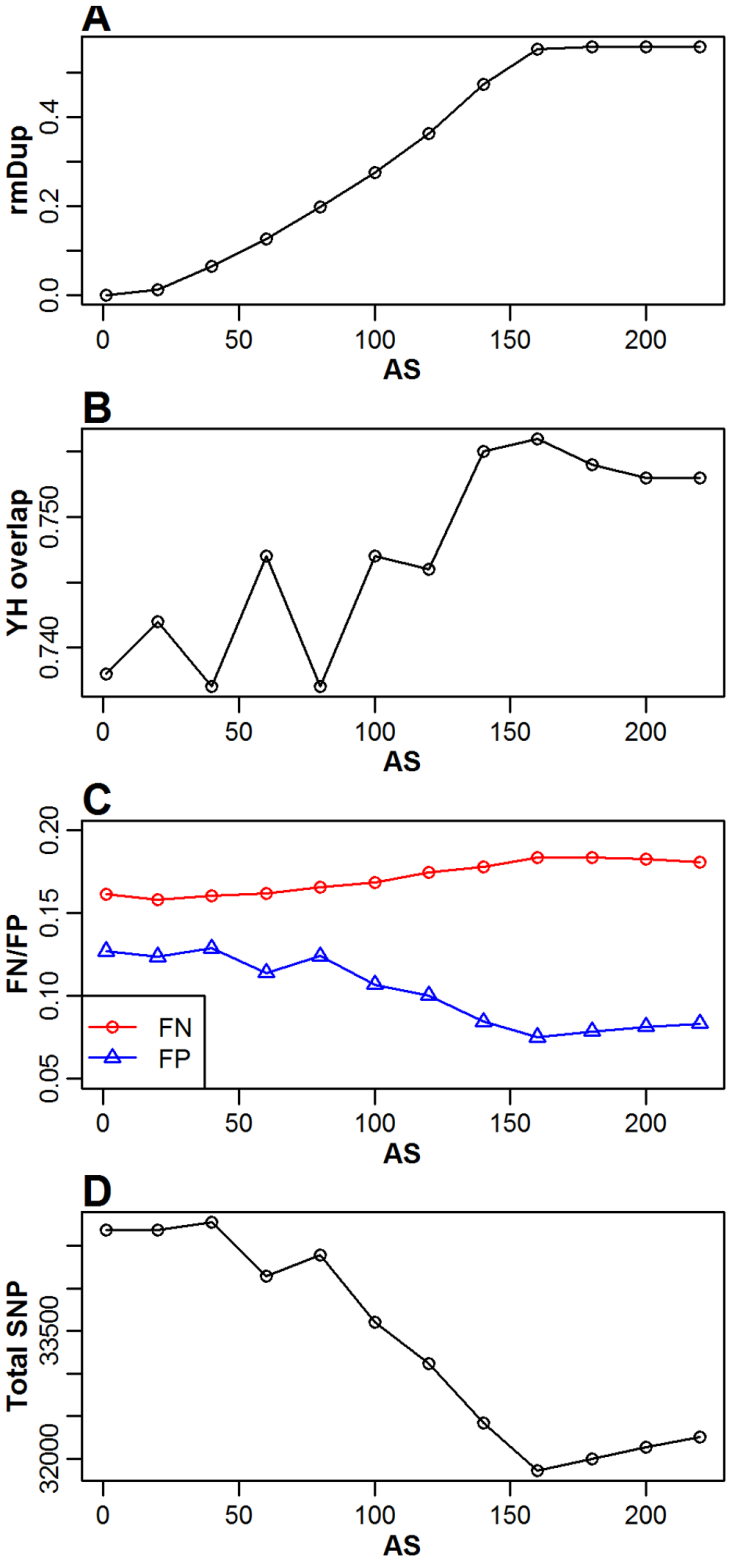
The relationship between the Alignment Score (AS) and duplication, YH-WGS overlap ratio, FN/FP ratio and total SNP number. (A) The duplication rate increases as the AS threshold increases, eventually reaching a stable level. (B) The percent of SNP overlaps with the YH-WGS. (C) The false negative rate and the false positive rate change with the AS threshold. (D) The total SNP number changes with the AS threshold.

We tested this method on sample TP00005. The duplication rate increased as the AS threshold increased, and this rate stabilizedat57% when the AS exceeded 160. Simultaneously, the false positive rate, total SNP number and overlap rate with YanHuang-Whole Genome Sequencing (YH-WGS) decreased (Figure 3). When the AS was higher than 100, many of the duplicates were removed. The remaining reads are therefore more reliable, indicating that the SNP accuracy is higher, the overlap rate is higher and the false positive rate is lower. In the OTG-snpcaller pipeline, the AS threshold is a user-controlled option. Higher AS will increase the SNP accuracy but decrease the depth. Therefore, if the user is concerned about a low depth covered region, he/she should set a lower AS threshold.

#### c. Alignment optimize structure (AOS) –reduce the SNP false negative (FN) result

As previously mentioned, the data generated by Proton has a high gapped reads ratio for homopolymer errors. When a candidate SNP site happens to be a gap site, it is difficult to distinguish between a SNP and an InDel, and even worse, it may not be detected at all.

Therefore, it is necessary to preprocess the data to filter out these inappropriate gaps before SNP calling, and for this purpose, we developed a filtering method named Alignment Optimize Structure (AOS). In this method, every base of the reference genome is examined. For each base position, if the base in the read is not concordant with the base in the reference, the following two steps are processed.

#### Step 1: Homopolymer confirmation

**Figure 4:**
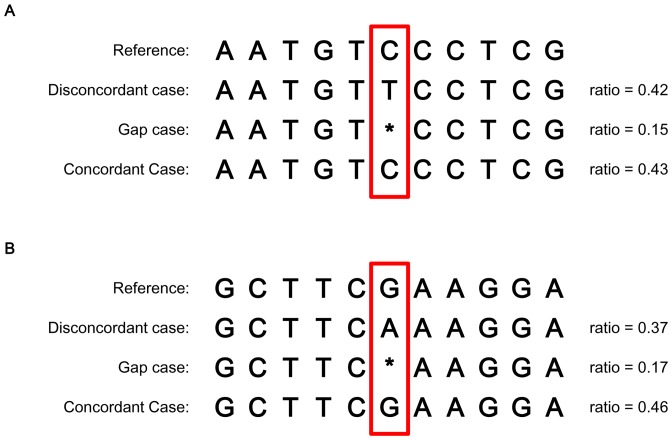
Neighboring bases pattern of a mismatch position. (A) 3-base-homopolymer neighboring bases appear at the reference. (B) 3-base-homopolymer neighboring bases appear at reads.

If the position has at least 3-base-homopolymer neighboring bases (including the base in question) in the reference (Figure 4A) or in the aligned reads (Figure 4B), proceed to Step 2. If not, the next base will be examined.

#### Step 2: Improper gap removal

Each position under examination is categorized as 1 of 3 cases: concordant with reference, discordant with reference and gap (Figure 4). Then, for all the reads covering this position, we calculated the ratio of each case. For the gap case, if the ratio is under 0.2 [13], we define those gaps as improper gaps and remove them from the alignment result.

#### d. GATK best practices

GATK performs well on Illumina data. It is common to use BWA for alignment, Picard to remove duplicates and GATK for variant calling. However, for Ion Torrent data, analysis with TMAP, RDAST, AOS and GATK is a better choice for variant calling. During the final GATK function, there are four steps to call variants: 1.local realignment, 2.base quality recalibration, 3. call variants and 4.variant quality recalibration [11], [14].

### 2. Data Summary

Three libraries of YH sample exon region data were analyzed using the OTG-snpcaller pipeline. Two libraries were captured using the Agilent SureSelect V3 kit (the target region covers approximately 50 Mb) and the other was captured by the NimbleGen SeqCap EZ V2 kit (the target region is approximately 44 Mb). Each library gets more than 60 M reads. In fact, the TP00005 library gets more than 90 M reads. The TP00006 library was sequenced in two runs (TP00006-1 and TP00006-3). For all libraries, the unique mapping rate exceeded 97% (Table 2). The capture specificity, which describes the proportion of reads overlapped with the target region, exceeded 75%. The coverage of the target region exceeded 95%. The mean depth of the target region exceeded 68X. For all sites in the target region, more than76% have a depth higher than 20X.

**Table 2:** Data summary of the 3 YH libraries.

Sample	Clean reads	Map rate	Duplication rate	Specificity	Mean depth	Coverage >1X	Coverage >20X
**TP00005**	93009491M	97.82%	41.70%	80.45%	94.21	97.43%	83.63%
**TP00006-1**	56867822M	97.58%	33.97%	80.87%	68.86	96.40%	77.16%
**TP00006-3**	75333828M	98.05%	33.33%	83.24%	99.7	96.85%	82.30%
**TP00010**	64369880M	97.80%	20.78%	75.38%	101.38	95.68%	78.98%

### 3. Evaluation of OTG-snpcaller

#### 3.1. OTG-snpcaller vs. TVC

**Figure 5:**
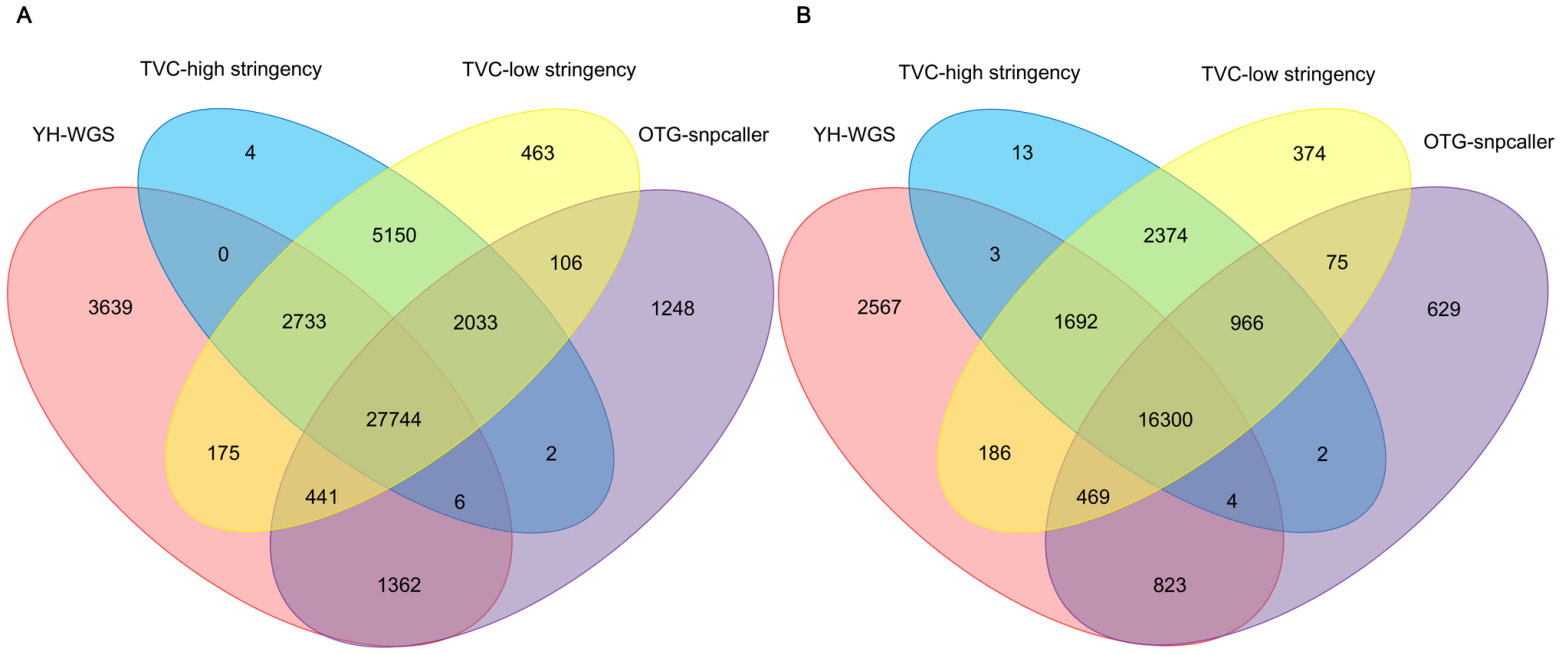
Venn diagrams of WGS, TVC high stringency, TVC low stringency and OTG-snpcaller. (A) Data from the TP00005 library. (B) Data from the TP00010 library. OTG-snpcaller exhibits more overlap (above 75%) with YH-WGS than both TVC high stringency and TVC low stringency.

Using the OTG-snpcaller method, we analyzed two libraries, TP00005 and TP00010, and compared the results with those of TVC (both high and low stringency parameters) (Figure 5). The TP00005 library was captured using the Agilent 50-Mb exome kit and TP00010 was captured by the NimbleGen 44-Mb exome kit. There were 32949 and 19268 SNPs detected using the OTG-snpcaller method, respectively. After using various methods for SNP call, we evaluated the False Positive (FP) and False Negative (FN) rates for each method. To evaluate FPs, we used the YH-WGS [15] data as the control. First, we filtered the YH-WGS SNPs within the exon target region. Then, we evaluated the concordance with our OTG-snpcaller result. We discovered that the FP rate of our OTG-snpcaller method was under 10%, which is much better than that of the TVC method (above 14%) (Table 3).

**Table 3:** Comparison of OTG-snpcaller and TVC.

	TP0005			TP00010		
Category	OTG-snpcaller	TVC-low stringency	TVC-high stringency	OTG-snpcaller	TVC-low stringency	TVC-high stringency
**Total SNP nunber**	32942	38854	37673	19268	22449	21358
**dbrate**	97.48%	96.46%	97.06%	97.92%	96.86%	97.68%
**Ti/Tv**	2.7018	2.5415	2.5886	3.0736	2.8401	2.9196
**Het/hom**	1.3	1.63	1.61	1.3	1.56	1.54
**dbSNP Ti/Tv**	2.7784	2.647	2.6639	3.1532	3.0017	3.0282
**FNrate**	**7.79%**	5.98%	7.41%	9.82%	7.48%	10.04%
**FPrate**	9.12%	18.80%	18.10%	7.50%	15.70%	14.70%
**Overlap with YH-WGS**	75.60%	71.60%	71.10%	75.00%	73.00%	71.50%
Consensus*	99.93%	99.91%	99.92%	99.93%	99.92%	99.93%

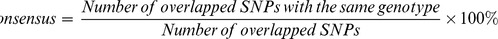

To assess the FN rate, we used the Illumina 1 M-Duo genotyping data as the control. First, we filtered the 1 M-Duo data with the target region bed files of the Agilent 50-Mb and NimbleGen 44-Mb kits to obtain the SNPs, which were located in the target region. Then, we compared the filtered SNPs with the OTG-snpcaller results to discover how many of the genotyping SNPs could be detected using our methods and therefore obtain the FN rate. Our results revealed that the FN rate of OTG-snpcaller was under 10%, which is comparable with that of TVC (Table 3).

#### 3.2. OTG-snpcaller vs. Hiseq pipeline

To further evaluate OTG-snpcaller, we compared our results with those of the YH-Hiseq data (PE90), which was sequenced using Hiseq2000 and analyzed using the Hiseq pipeline (BWA, Picard and GATK). Using the same clean data (6G bases), we found that the results of these two platforms were comparable with the exception of the duplication rate (Table 4). YH-Hiseq has a higher mean depth than did TP00005-cut(random extraction of 6G bases from TP00005), whereas the two libraries exhibited nearly the same coverage rate of the target region for at least 20X. YH-Hiseq detected1000 more SNPs than did TP00005-cut. The dbSNP rate, the YH-WGS overlap rate and the Ti/Tv are equivalent. Notably, TP00005-cut has a higher FN rate and a lower FP rate than does YH-Hiseq.

**Table 4:** Comparison of the OTG-snpcaller and Hiseq pipelines.

Category	TP00005-cut	YH-Hiseq
**Clean data**	6978515693	6978515760
**Map rates**	97.83%	98.83%
**Duplication rate**	35.69%	12.33%
**Capture specificity**	80.81%	75.07%
**Target coverage**	96.96%	97.70%
**Target mean depth**	67.93	72.04
**Coverage of target at least 20X**	79.20%	78.60%
**Total SNP number**	31365	32891
**db rate**	97.97%	99.10%
**Ti/Tv**	2.7402	2.6857
**FN rate**	9.52%	9.11%
**FP rate**	7.92%	10.19%
**Overlap with YH-WGS**	73.70%	74.60%
**Consensus**	99.93%	99.93%

**Figure 6:**
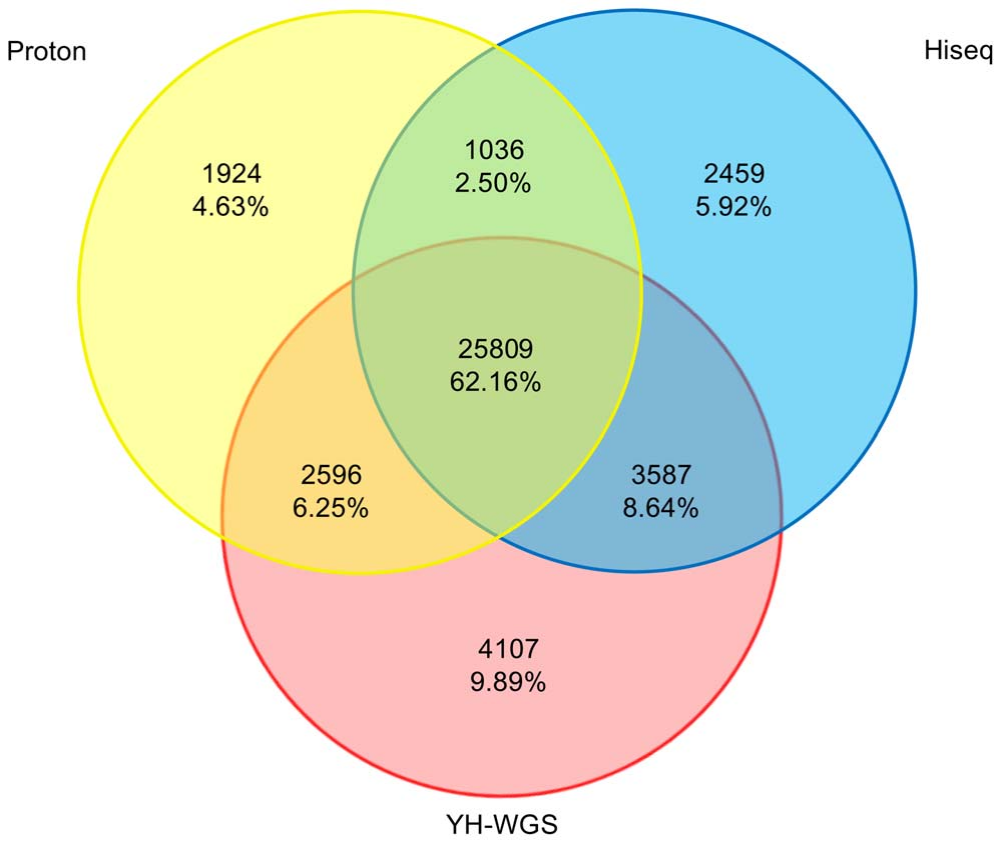
Venn diagram of the overlap in the numbers of variant calls using Proton and Hiseq.

For further analysis, both the Proton and the Hiseq predicted variant lists were compared to the YH-WGS dataset. Overall, the overlap SNPs across the three datasets included 25809 results (62.2%). **Figure 6** shows a Venn diagram of the overlap among all three methods. There are 28406 (73.7%) and 29397 (74.6%) SNPs for Proton and Hiseq, respectively, that also overlap with YH-WGS. There are several origins for the platform-specific SNPs, the false positives and the false negatives for YH-WGS (the sequencing depth of YH-WGS data were only approximately 30X, so some SNPs may have been missed) and the various generations of cell lines.

### 4. Performance Comparison of OTG-snpcaller with and without Optimization

RDAST and AOS are crucial parts of OTG-snpcaller. To verify the contributions of our RDAST and AOS algorithms, we formed four combinations of algorithms to analyze one TP00005library (Table 5). The first algorithm is OTG-snpcaller, which is the standard OTG-snpcaller method (TMAP, followed by RDAST, by AOS, and by GATK). The second is OTG-snpcallernoDup, which excludes the RDAST method (TMAP, followed by AOS and GATK). The third is OTG-snpcallernoAOS, which excludes the AOS method (TMAP, followed by RDAST and GATK). The fourth is OTG-snpcallernoBoth, which excludes both the RDAST and AOS methods (TMAP followed by GATK). After RDAST treatment, the specificity increased from 85.88% (OTG-snpcallernoDup) to 90.88% (OTG-snpcaller). Conversely, the sensitivity also increased 5%, from 87.03% (OTG-snpcallernoAOS) to 92.21% (OTG-snpcaller) after treatment with AOS. Moreover, the sensitivity and specificity increased substantially after treatment with RDAST and AOS. Meanwhile, the overlap with WGS increased significantly, and the consensus with the genotyping data remained close to 99.92% (Table 5).

**Table 5:** The performance comparison of OTG-snpcaller with OTG-snpcallernoDup (no RDAST), OTG-snpcallernoAOS (noAOS), and OTG-snpcallernoBoth (no RDAST or AOS).

Method	Total SNP number	dbsnp rate	Sensitivity*	Specificity**	Overlap with YH-WGS	Consensus
**OTG-snpcaller**	32942	97.48%	92.21%	90.88%	75.60%	99.93%
**OTG-snpcallernoDup**	35419	93.23%	92.62%	85.88%	73.00%	99.92%
**OTG-snpcallernoAOS**	30848	98.01%	87.03%	91.41%	71.80%	99.94%
**OTG-snpcallernoBoth**	32371	94.04%	86.08%	86.76%	68.60%	99.93%

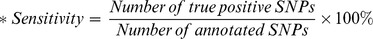

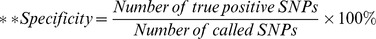

**Figure 7:**
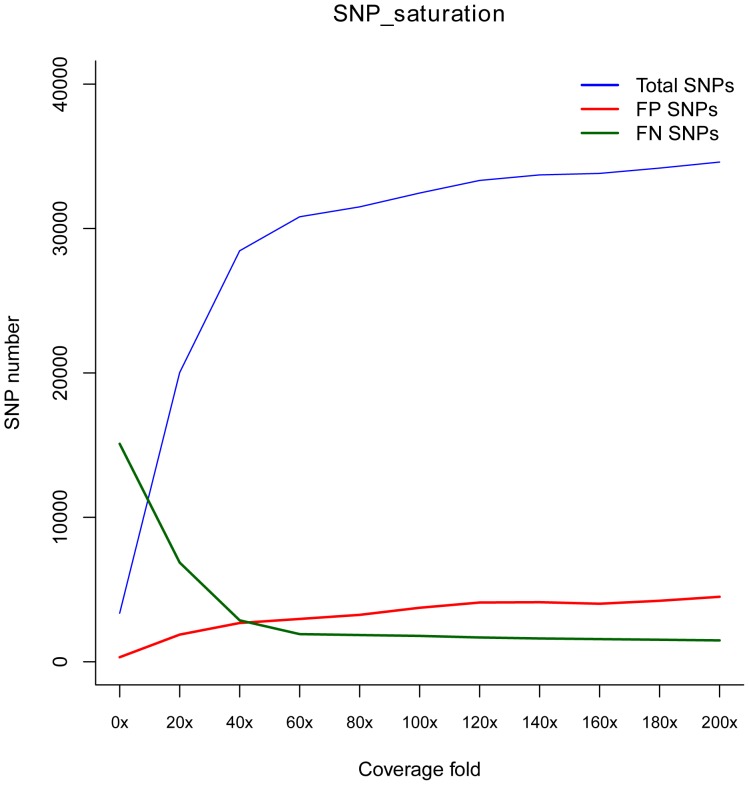
The analysis of total SNP, FN and FP under coverage folds.

We merged the TP00006-1 library with the TP00006-3 library using Picard MergeSamFiles to obtain enough data (depth of 200X) for SNP saturation statistics. By randomly extracting the data, we produced an image of the SNP number that varied with the depth from 0X to 200X. As shown in **Figure 7**, 0–40X is a rapid growth period. With the increase in sequencing depth, both the total SNP number and the FPs grew rapidly, and the FNs markedly decreased. Moreover, 40–100X is a slow growth period during which the change of all indicators tends to slow. However, 100–200X is a plateau period, during which the performance of OTG-snpcaller peaks. At this point, the method has the most total SNPs with lowest FP SNP rate, but it would be difficult to reduce the number of FN SNPs. Therefore, users can choose an appropriate and cost-saving approach that takes their project and the budget into account for Proton sequencing.

## Discussion

Herein, we have developed a pipeline for SNP calling of Ion Torrent data called OTG-snpcaller, which focuses on managing the duplicates and the gaps generated by Ion Torrent sequencing. During target region SNP detection, we combined TMAP with GATK. Our special duplicate removal (RDAST) and alignment results optimization (AOS) procedures improved the accuracy, sensitivity and specificity of SNP detection.

According to the read alignments generated by the Integrative Genomics Viewer (IGV) [16], [17], we identified three main reasons for false negative SNP results. The first is that the detection area was not covered. The second is that the base quality was too low, and the third is that the proportion of gaps was too high, for example, above 0.5. The use of our AOS is unable to debug this case.

Although OTG-snpcaller was first designed for the exon regions from Proton sequence data analysis, it is also appropriate for Proton whole genome sequence (WGS) data. For the other applications of the Ion Torrent sequence platform, such as the Cancer Panel (sequenced by PGM) and the PGD (Preimplantation Genetic Diagnosis), it is also instructive. With the upgrade of Proton sequence chips and the torrent sever base calling, OTG-snpcaller will perform better with respect to SNP detection accuracy.

## Materials and Methods

The YH cell line genomics DNA was used to construct the library. TargetSeq exome capture libraries were constructed according to “Ion TargetSeq Exome Enrichment for the Ion Proton System” and “SureSelect Target Enrichment System” with small modifications.

For each sample, 1 µg of DNA was sheared using Covaris S2 to an average size of 150bp.

Then, the sheared DNA was end-repaired, ligated with Ion adaptors, size selected, nick translated and PCR amplified. Both 500 ng and 1 µg of the Ion library was used for exome capture using the Agilent SureSelect Exome 50-Mb kit and the NimbleGen SeqCap 44-Mb Exome kit according to the standard protocols. The captured library was then quantitatively analyzed on the Agilent Bioanalyzer. Finally, the captured DNA library was processed for Proton sequencing.

After sequencing, the bam file produced by Proton was mapped against to the HG19 reference using the TMAP3.6 software with default parameters. For TargetSeq, the region bed file was necessary for the basic bioinformatics analysis. Moreover, the alignment needed to be filtered using the RDAST program with an AS score of 160 to reduce PCR errors and optimized using AOS with a gap ratio 0.2 to reduce the false negative results. Both of these two parameters had important effects on the sensitivity and specificity of variant detection. After all these steps, we choose GATK for SNP call (see http://www.broadinstitute.org/gatk/guide/best-practices for more information about GATK best practices). For further evaluation, ANNOVAR [18] annotation was applied to provide more information associated with the SNP database and the functional region in the genome.

### Data Accession

The Proton and Hiseq sequence files have been submitted to the NCBI Sequence Read Archive under accession no. SRP040322.
