# Correction: OTG-snpcaller: An Optimized Pipeline Based on TMAP and GATK for SNP Calling from Ion Torrent Data

**DOI:** 10.1371/journal.pone.0138824

**Published:** 2015-09-16

**Authors:** Pengyuan Zhu, Lingyu He, Yaqiao Li, Wenpan Huang, Feng Xi, Lin Lin, Qihuan Zhi, Wenwei Zhang, Y. Tom Tang, Chunyu Geng, Zhiyuan Lu, Xun Xu

The Data Accession section of this paper is incorrect. The section should say: The authors confirm that all data underlying the findings are fully available without restriction. All relevant data are available from Figshare: http://dx.doi.org/10.6084/m9.figshare.1509777.

## References

[1] Zhu P, He L, Li Y, Huang W, Xi F, Lin L, et al (2014) OTG-snpcaller: An Optimized Pipeline Based on TMAP and GATK for SNP Calling from Ion Torrent Data. PLoS ONE 9(5): e97507 doi:10.1371/journal.pone.0097507 24824529 10.1371/journal.pone.0097507 PMC4019570

